# Transcriptomics of the late gestation ovine fetal brain: modeling the co-expression of immune marker genes

**DOI:** 10.1186/1471-2164-15-1001

**Published:** 2014-11-19

**Authors:** Maria B Rabaglino, Maureen Keller-Wood, Charles E Wood

**Affiliations:** Departamento de Reproducción Animal, Fac. Agronomía y Veterinaria, Universidad Nacional de Río Cuarto, Córdoba, Argentina; Department of Pharmacodynamics, College of Pharmacy, University of Florida, Gainesville, FL 32611 USA; Department of Physiology and Functional Genomics, College of Medicine, University of Florida, Gainesville, FL 32611 USA

## Abstract

**Background:**

Major changes in gene expression occur in the fetal brain to modulate the function of this organ postnatally. Thus, factors can alter the genomics of the fetal brain, predisposing to neurological disorders later in life. We hypothesized that the physiological dynamics of the immune system transcriptome of the fetal brain during the last stage of gestation will reveal patterns of immune function and development in the developing brain. In this study we applied weighted gene co-expression analysis of microarrays performed on ovine fetal brain samples, to model the changes in gene expression throughout the second half of gestation.

**Results:**

Clusters of co-expressed genes that strongly increase in expression toward the first day of extra-uterine life are related to the hematopoietic lineage, while activation of immune pathways is induced after birth. Moreover, the pattern of gene expression suggests induction of tolerance mechanisms, probably necessary to protect highly produced proteins –such as myelin basic protein- from an autoimmune attack.

**Conclusions:**

This study provides insight into the dramatic changes in gene expression that take place in the brain during the fetal life, especially during the last stage of gestation, and suggests that the immune system may have an important role in maturation of the fetal brain, which if disrupted or altered, could have negative consequences in postnatal life.

**Electronic supplementary material:**

The online version of this article (doi:10.1186/1471-2164-15-1001) contains supplementary material, which is available to authorized users.

## Background

Normal brain development requires temporal changes in gene expression. In the human brain, the major changes in spatio-temporal gene expression occur during the late gestation fetal life [1]. Little is known about development of immune function in the fetal brain, although there is a growing literature that some chronic diseases of the adult, such as hypertension [2], schizophrenia [3], and autoimmune disorders [4] are at least in part the result of immune dysfunction within the brain. Several studies have analyzed the effect of diverse stimuli (such as nutrition, stress, toxics and so on) on the activity of the immune system in the fetal brain and the repercussions in adulthood (reviewed in [5, 6]). Despite its importance, the ontogeny of immune cells within the fetal brain is not fully understood. The focus of studies on immune development in late gestation has been on development of these cells in somatic sites (i.e., liver, thymus, spleen, etc.). However, a better understanding of the molecular development of the immune system within the brain holds promise for design of novel therapeutics that can redirect the “programmed” brain back to a normal trajectory of development. The purpose of the present study is to model changes and coherence of gene expression in cerebral cortex, brainstem, hippocampus, and hypothalamus in order to obtain a broad view of brain ontogeny throughout the second half of gestation. We found, in addition to expected changes in pathways related to brain structure and metabolism, that there was an over-representation of genes in the hematopoietic pathway. We therefore modeled the changes in genes in the hematopoietic pathway over this period of ontogeny. We expect that the study of genomics related to immune system development in late gestation fetal brain might reveal molecular mechanisms of neuronal protection. Moreover, investigation of the genomics of fetal brain development could provide a better understanding of mechanisms underpinning postnatal neurological disorders.

We used the sheep as an animal model to identify co-expressed genes in the above regions of the ovine fetal brain, from mid-gestation (80 days, n = 4; 100 days, n = 4; 120 days, n = 4; 130 days, n = 4; 145 days, n = 4) to one day of postnatal life (n = 4). In the ewe, gestation length averages 147 days. The ovine fetus is an excellent model to study brain development since the entire gestational equivalent of human brain development occurs *in utero* [7]. Altricial species (such as rats and mice) are not suitable models for human brain development because of the marked immaturity of the brain at birth in these species. Using a newly-available ovine array and weighted gene co-expression network analysis (WGCNA) on differentially expressed genes (Additional file 1: Figure S1), we identified co-expressed genes in different regions of the ovine fetal brain, from mid-gestation to one day of postnatal life. WGCNA determines pair-wise correlations between gene expression profiles to create modules (clusters) of co-expressed genes (Additional file 2: Figure S2). The resulting modules for each network were related with gestational age to determine gene significance (GS, correlation of *i*-th gene with the temporal pattern) and module membership (MM; correlation of the *i-*th gene with respect to its corresponding module) for each gene (Additional file 3: Table S1 and Additional file 4: Figure S3). Top modules were defined as those having the highest positive or negative correlation between GS and MM; i.e., modules with highest increasing or decreasing expression pattern from mid-gestation to 1 day of extra-uterine life (Additional file 5: Figure S4). These modules (or gene clusters) were selected for further analyses.

## Results and discussion

### Enrichment and network analysis

Significantly enriched Biological Processes (BP; p < 0.01; WebGestalt software [8]) of the positively correlated top modules from each brain region are shown in Additional file 6: Table S2. Negatively correlated top modules were mainly enriched with cell cycle. In agreement with other studies [1, 9], groups of co-expressed genes that increase in expression toward the end of gestation are related with response to stress and stimulus, myelination, apoptosis, generation of energy, and angiogenesis, while genes that decrease in expression are mainly related to cell cycle.

These events occur because during the last steps of fetal brain development there is a deceleration in cell division (quiescence) simultaneous with an acceleration in neuronal developmental and differentiation [9–11].

**Figure 1:**
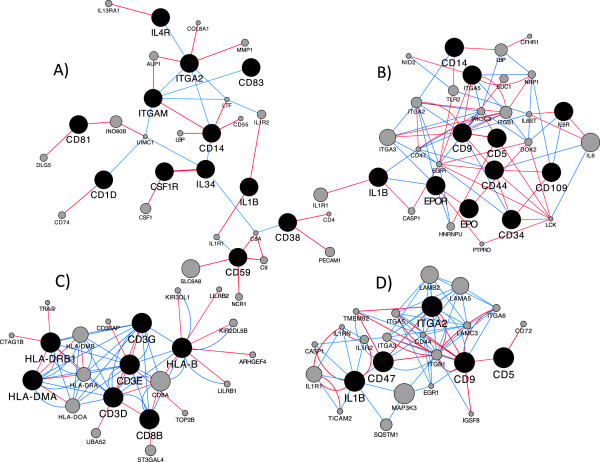
**Networks composed by nodes (genes) related to the hematopoiesis in the ovine fetal brain.** Black nodes are genes belonging to the top positively correlated module for each brain region. Grey nodes are genes inferred by the GeneMania plugin. Brain regions are cortex **(A)**, brainsteam **(B)**, hippocampus **(C)** and hypothalamus **(D)**.

**Figure 2:**
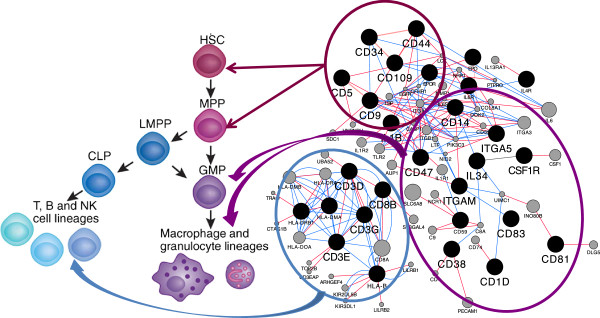
**The nodes (genes) of the merged network represent markers for immune cells of the hematopoietic lineage.** HSC, Hematopoietic stem cell; MPP, multipotent progenitor; LMPP, lymphoid-primed multipotent progenitor; CLP, common lymphoid progenitor; GMP, granulocyte macrophage progenitor. Hematopoiesis figure reprinted with permission from the Nature Publishing Group. (Source: Welinder and Murre, 2011. Ldb1, a new guardian of hematopoietic stem cell maintenance. Nature Immunology, Vol 12:2. Figure 1, page 113).

Significantly enriched KEGG pathways (p < 0.01; WebGestalt software [8]) were compared to identify common enriched pathways between all brain regions (Table 1). Some of the common enriched pathways in the positively correlated top modules were expected in the developing fetal brain, such as those related with metabolism. Genes related with endocytosis and lysosomal activity were also expected to be expressed at increasing levels by the end of gestation, since they are particularly relevant in polarized cells like the neurons [12]. Notably, a common enriched KEGG pathway related with the immune function was the hematopoietic cell lineage. We focused further analysis on genes related with the hematopoietic lineage. These genes were visualized as separate networks, according to the brain region, using the Cytoscape software (3.0) [13] and physical and genetic interactions between genes were inferred by use of the GeneMania plugin [14] (Figure 1). Then, these networks were merged together to obtain a single network connecting the nodes corresponding to the genes of interest from the four brain regions. The nodes (genes) that comprise this new network represent markers for different immune cells and at different stages of maturation. Therefore, the whole network can be correlated with the hematopoietic lineage (Figure 2), as was indicated by the “hematopoietic cell lineage” as a common enriched KEGG pathway.

**Table 1:** **Significantly enriched KEGG pathways in positive correlated top modules from all fetal brain regions**

	Adjusted p-values			
KEGG Pathway	Cortex	Brainstem	Hippocampus	Hypothalamus
Endocytosis	2.42E-10	5.68E-08	0.0006	0.0013
Hematopoietic cell lineage	0.0027	0.0026	0.0002	0.0098
Insulin signaling pathway	3.11E-08	0.0003	0.0007	0.0016
Lysosome	7.19E-16	8.09E-08	0.0005	0.0001
MAPK signaling pathway	9.06E-09	0.0012	0.0006	0.0002
Metabolic pathways	7.12E-36	3.47E-26	0.0001	5.40E-16
Phagosome	2.37E-06	1.07E-07	6.00E-06	0.0004
Tight junction	5.29E-05	0.0014	0.0006	0.0004

These results do not necessarily mean that the brain is a hematopoietic organ similar to liver or spleen during fetal life. Nor can it be concluded from this study that hematopoietic stem cells are present in the ovine fetal brain. Rather, these results strongly suggest the presence of developing myeloid and lymphoid derived cells in different regions of the fetal brain (*vide infra*).

### Detailed analysis of immune marker genes

Marker genes for progenitor immune cells were found in the top positive correlated module of the ovine fetal brainstem. These markers are CD34, CD109 CD44, CD5 and CD9 (Additional file 7: Figure S5). CD109 antigen is expressed by a subpopulation of CD34+ Hematopoietic stem cell (HSC) and progenitor cells in the fetal and adult bone marrow [15]. An isoform of CD44 is expressed on CD34+ cells: HSC and progenitor cells [16]. Progenitors cells of the myeloid lineage, such as colony-forming unit macrophage/dendritic cells, express the colony stimulating factor 1 receptor (CSF1R) for differentiation. The resident myeloid inflammatory cells of the central nervous system (CNS) parenchyma are microglial cells. Thus, expression of CSF1R seems critical for microglia development. In mice, the absence of CSF1R (Csf1r ^-^/^-^) results in essentially no macrophages/microglia (<99% depletion) in the embryonic and early post-natal brain [17]. In mice, interleukin 34 (IL34) binds to the CSF1R receptor with high affinity to regulate myeloid development and it can substitute for colony stimulating factor 1 (CSF-1) *in vivo*. IL34 is more highly expressed than CSF-1 mRNA in most of the regions of the developing brain [18]. These genes were part of the top positive correlated module in the cortex network, as well as other genes markers of antigen presenting cells, including CD1D, integrin, alpha M (CD11B) and CD81, (Additional file 8: Figure S6) indicating the development of microglia in the fetal CNS parenchyma.

**Figure 3:**
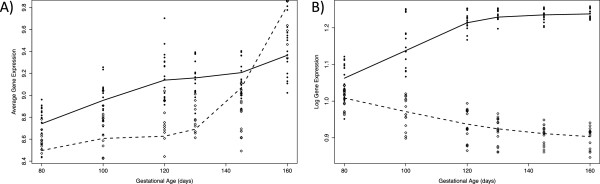
**Trajectories of gene expression for immune related genes. (A)** Genes related with the hematopoietic lineage are increased in expression along fetal age and first day of extrauterine life –day 160-(solid line, filled circles). Genes related with immune system activation are strongly induced after birth (dashed line, open diamond). **(B)** Opposite trajectory expression of CD24 gene (dashed line, open diamond) and MBP gene (solid line, filled circle).

Interestingly, genes related to hematopoiesis increased in expression during fetal life, but genes related to immune system activation and antigen presentation are strongly induced after birth (Figure 3A). These genes are those encoding for the mayor histocompatibility complex class I and II; such as HLA-B and B2M (class I) or CD74, HLA-DMA and HLA-DRB1 (class II), or genes encoding for components of the immunoproteosome necessary to process class I MHC peptides, such as PSMB8 and PSMB10.

Therefore, these results suggest a regulated development of the immune system in the fetal brain. In agreement with this concept, mRNA expression of genes markers for mature T-cells including the three chains of the CD3 marker (CD3G-CD3E-CD3D) were strongly increased after birth in the ovine fetal hippocampus, when measured by RT-PCR (Additional file 9: Figure S7), rising the possibility that T cells are present in the fetal brain.

Circulating lymphoid cells could trespass the relatively permeable blood brain barrier (BBB) during fetal life and reach the brain parenchyma [19]. If any of these cells escape central tolerance, it is possible for them to react against highly antigenic self-proteins which are produced at increasing rate in late gestation, such as the highly antigenic myelin basic protein (MBP) causing the autoimmune demyelinating disease of the CNS known as multiple sclerosis or in the animal model, experimental autoimmune encephalitis (EAE) [20].

Expression of MBP gene was strongly positively correlated with fetal age for all brain regions. Correlations ranged from 0.87 in hippocampus to 0.92 in cortex, with an average of 0.893. The gene encoding for MBP was one of the top positive correlated modules for all brain regions but also showed a strong increasing mRNA expression measured by RT-PCR in the ovine fetal cortex (Additional file 10: Figure S8A). Consequently, resident cells of the CNS should be able to counteract the action of potential auto-reactive lymphocytes.

Remarkably, an immune marker gene that was part of the top negatively correlated modules for all brain regions was CD24. Correlations of this gene with fetal age ranged from -0.83 in hippocampus to -0.98 in cortex (average -0.895). Thus, the expression trajectory for this gene was the opposite of MBP (Figure 3B). mRNA expression of this gene, as measured by RT-PCR in ovine fetal cortex was dramatically decreased with fetal age (Additional file 10: Figure S8B). CD24 is a surface glycoprotein that is strongly expressed by immature B- and T- lymphocytes, but which disappears from T cell surface as T cell mature. However, activation of T cells resulted in a rapid induction of CD24 expression [21]. CD24 is also expressed on resident cells of the CNS [22] and it is able to inhibit the proliferation of immature neurons [23].

Expression of CD24 in both T cells and non-T host cells is required for the development of EAE [24]. Expression of CD24 in T cells actively inhibits clonal deletion (necessary for central tolerance) allowing the expansion of autoreactive T cells [25]. Expression of CD24 in the CNS resident cells, including microglial cells and astrocytes, costimulates myelin antigen specific T cells and promotes their effector function, promoting the autoimmune inflammation in the CNS [26]. Taken together, these data imply that CD24 is involved in the autoimmune reaction to myelin antigens.

Microglia cells are able to acquire a dendritic cell phenotype and mediate peripheral tolerance to avoid progression of EAE [27]. Moreover, protection from EAE development, mediated by dendritic cells, was exclusively associated with low expression of CD24, when compared to EAE controls [28]. Thus, we speculated that the strong downregulation of CD24 with advancing fetal age results in a tolerance mechanism necessary to avoid an autoimmune reaction to myelin antigens, such as MBP, which could be related to the susceptibility to this type of disease later in life.

Accordingly, genes related with immune tolerance followed a pattern of increasing expression in late gestation. The Fc fragment of IgG, low affinity IIb, receptor (FCGRIIB) for example, is an inhibitory receptor on myeloid cells that can regulate T cell tolerance and promotes T-regulatory cell induction by increasing interleukin 10 (IL10) production [29, 30]. Both IL10 and transforming growth factor, beta 1 (TGFb), another cytokine involved in T-regulatory cell induction, showed an increased expression in the cortex with fetal age as measured by microarray and qRT-PCR (Additional file 10: Figure S8C).

## Conclusions

This study provides insight into the dramatic changes in gene expression that take place in the brain during the fetal life, especially during the last stage of gestation.

Results from the present study propose a regulated development of the immune system in the ovine fetal brain. This concept implies induction of tolerance mechanisms, to protect fetal myelin from a possible autoimmune attack, and activation of the immune response after birth, which could be part of the physiological adaptation of the newborn for the extrauterine life. These findings could provide the basis for new investigation in this area, in order to understand key regulators of fetal brain development.

## Methods

### Tissue collection

Tissues were collected from fetuses at 80 (80 d, *n* = 4), 96–100 (100 d, *n =* 4), 120 (120 d, *n* = 4), 130 (130 d, *n* = 4), and 142–144 (145 d, *n =* 4) days of gestation and on the first (1 d, *n =* 4) day after delivery. Each group included one set of twin fetuses. None of the ewes showed any signs of impending labor. For collection of fetal tissues, ewes were killed with 20 ml of Euthasol solution (7.8 g pentobarbital and 1 g phenytoin sodium; Virbac AH, Fort Worth, TX) administered intravenously, the fetus was quickly removed, and the fetal brain was removed. Brain dissection was performed immediately, using sterile instruments and gloves. First, the head was separated from the backbone cutting cranial to the axis of the vertebrae. Second, the posterior cranial bones were separated using forceps. With a scalpel we cut the pituitary stalk and optic nerves to release the brain, which was then deposited onto a sterile container or surface. Holding the brain upside down, the hypothalamus was dissected, followed by both hippocampi and the brainstem. Finally, a section of the frontal cortex was dissected. Fetal tissues were rapidly frozen in liquid nitrogen and stored at -80°C.

The use of animals in this project was approved by the University of Florida Institutional Animal Care and Use Committee.

### RNA extraction and preparation

RNA was extracted from the cortex, hippocampus, hypothalamus and brainstem using Trizol (Invitrogen, Carlsbad, CA) and following the manufacturer’s directions. The RNA was resuspended in RNAsecure, and stored at -80°C in aliquots until use. For microarray analysis, 20 ug of RNA was DNase treated using the Turbo RNase-free DNase kit (Ambion, Foster City, CA), the concentration determined with a Nanodrop spectrophotometer (ND-1000, ThermoFisher, Wilmington DE) and the integrity of the RNA was measured using an Agilent Bioanalyzer, 2100 model. One μg of the DNase-treated RNA was labeled with Cyanine 3 (Cy3) CTP with the Agilent Quick Amp kit (5190–0442, New Castle, DE) according to their methodology, purified with the Qiagen RNeasy kit (Valencia, CA) as according to Agilent’s revision of the Qiagen protocol as shown in the Quick Amp kit protocol except that the microcentrifugation spins were performed at room temperature instead of 4°C. The resulting labeled cRNA was analyzed with the Nanodrop spectrophotometer, and the specific activities and the yields of the cRNAs were calculated. The labeled cRNA was stored at -80°C until use.

### Microarray hybridization

This was performed following protocols from Agilent. Briefly, 600 ng of each labeled cRNA was fragmented and then mixed with hybridization buffer using the Agilent gene expression hybridization kit. These were applied to sheep 8 × 15 K array slides (Agilent 019921), containing 8 arrays with 15,208 oligomers with a length of 60 bases and hybridized at 65°C for 17 h at 10 rpm. A total of 3 slides (24 arrays) were employed per each brain region (six gestational ages times four replicates per gestational age). The arrays were washed, dried, stabilized, and scanned with an Agilent G2505B 2 dye scanner at the Interdisciplinary Center for Biotechnology Research at the University of Florida. Features were extracted with Agilent Feature extraction 9.1 software.

### Microarray data

The limma package was employed to import the raw data into R (http://www.r-project.org), perform background correction and normalize the data using the quantile normalization method [31]. Control probes and low expressed probes were filtered out, retaining for further analysis the probes that were at least 10% brighter than the negative controls on at least four arrays.

### Statistical analysis

The Bayesian Estimation of Temporal Regulation (BETR) algorithm was used to identify the differentially expressed genes (DEG) for each brain region at a False Discovery Rate (FDR) < 0.05. The first gestational age (80 days) was considered as baseline measurement and compared to the subsequent time points, to correlate the differential expression between time points. The algorithm was applied using the BETR package for R software. A detailed explanation of the mathematical model can be found in Aryee et al. [32]. This method returns the probabilities of differential expression for each gene in the data set. Genes with a probability higher than 99.99% were considered as DEG.

### Supervised weighted gene co-expression network analysis (WGCNA)

The DEG for each brain region was subjected to signed-WGCNA. Rows of each data set were collapsed forming an average expression of the genes with the same official symbol, in order to obtain unique identifiers for each gene in the working data set. The automatic method was employed for block-wise network construction and module detection. The co-expression similarity was raised to a soft thresholding power (β) of 12 to calculate adjacency. The adjacency for the signed network is defined as *a*_*ij*_ = |(1 + cor(*x*_*i*_*,x*_*j*_))/2|^β^ [33]. The resulting modules for each network were related with the gestational age to identify modules, or clusters, of co-expressed genes with increasing or decreasing expression pattern. Gene significance (GS) was defined as the correlation of *i*-th gene with a temporal pattern. Module membership (MM) was defined as the correlation of the *i-*th gene respects its corresponding module (the higher is the MM the more connected is the *i-*th gene with the other genes of the corresponding modules). The correlation coefficient of MM and GS was measure for each module, plotting MM versus GS. Higher correlation between MM and GS means that genes that are highly associated with the temporal pattern are also the central elements of the given module [33] (Additional file 3: Table S1). Modules that showed the highest correlation between MM and GS, either with increasing pattern (positive GS) or decreasing pattern (negative GS), were selected for Enrichment analysis. The top positively correlated module for each brain region was: the turquoise module for cortex and brainstem, the brown module for hippocampus and the blue module for hypothalamus. The top negatively correlated module for each brain region was: the blue module for cortex and brainstem, the yellow module for hippocampus and the turquoise module for hypothalamus (Additional file 4: Figure S3).

All the analyses were performed with the WGCNA package for R software. More details about the methodology for WGNA for network construction can be found at: http://labs.genetics.ucla.edu/horvath/CoexpressionNetwork/Rpackages/WGCNA/.

### Enrichment analysis

The bioinformatics tool applied for this purpose was WebGestalt (WEB-based GEne SeT AnaLysis Toolkit). This program is designed for functional genomic studies that generate large number of gene list. Then, it organizes the large set of genes based on common functional features, like GO categories or biochemical pathways [8]. The list of human official symbols for the genes composing the top modules for each network was submitted for enrichment biological processes and KEGG pathway analysis, selecting H. Sapiens as the organism of interest. The statistical method employed was the hypergeometric test, adjusting the p-values for Benjamini & Hochberg method, selecting the minimun of 5 genes per category and using the human genome as reference set. Significantly enriched Kegg pathways (p < 0.01) were compared to identify common enriched pathways between all brain regions.

### Network analysis

A gene network was constructed for each brain region using CytoScape version 3.0 [13] through the GeneMania plugin [14], which was used to infer network data. The set of functional association data between genes was downloaded from the Homo sapiens database. The list of human official symbols for the genes of interest was input into the GeneMania plugin to retrieve the corresponding association network. The association data employed was protein-protein and protein-DNA interaction. Then, the resulting networks were merged together using the Merge Networks tool of Cytoscape 3.0.

### Quantitative Real-Time (qRT)-PCR Validation

The mRNA samples extracted from the four brain regions at the six different gestational ages (4 fetuses/gestational age; 96 samples in total) were converted to cDNA with a High Capacity cDNA Archive kit using the methodology recommended by the kit manufacturer (Applied Biosystems, Foster City, Calif., USA). The newly synthesized cDNA was stored at -20°C until qRT-PCR was performed.

A total of 18 genes were selected for further validation by qRT-PCR. The cDNA from corresponding brain region was used to measure the mRNA of the following genes: Brainstem: CD34, CD109, CD44, CD5 and CD9; Hippocampus: CD3 gamma (CD3G), CD3 delta (C3D) and CD3 epsilon (CD3E); Cortex: CSF-1 CSF1R, IL34, CD11B, CD81, FCGRIIB, IL10, TGFB, CD24 and MBP.

Relative expression of selected genes were determined using primers (Sigma-Aldrich, St Louis, MO) and Sybr Green PCR Master Mix (Applied Biosystems, Foster City, CA). Primers were designed with Primer Express software (Applied Biosystems. Primers for CD109, CD44, CSF1, CSF1R, IL34, CD24 and MBP were designed from the corresponding bovine mRNA. Primers for CD34, CD5, CD9, CD3G, CD3D, CD3E, CD81, CD11B, FCGR2B, IL10 and TGFB were designed from the ovine mRNA. Primers sequences and accession numbers are reported in Additional file 11: Table S3. All primer pairs had efficiencies greater than 95%. The abundance of B-actin mRNA was determined in each brain region sample, using primers and VIC Taqman probes designed from the ovine B-actin sequence and Taqman qRT-PCR master mix (Applied Biosystems, Foster City, CA). All samples were run in triplicate for each gene and for B-actin. Relative mRNA expression of each gene was calculated by determining change in threshold cycle (ΔCt) between the mean Ct for each gene and the mean Ct for B-actin mRNA from the same sample. The effect of gestational age on each gene was analyzed by ANOVA using the ΔCt values. Pairwise comparisons among gestational age means were done by Duncan’s procedure. Data were graphed as the mean fold change in mRNA relative to the 80d group; fold change in each sample was calculated as 2^-ΔΔCt^, where ΔΔCt is the difference between ΔCt in each sample and the mean ΔCt in the control group. For all statistical analyses, the criterion for achieving statistical significance was P < 0.05.

## Electronic supplementary material

Additional file 1: Figure S1: Venn Diagram of the number of differentially expressed genes (DEG) in the ovine fetal brain. DEG were selected as those following a temporal profile during the last stage of gestation in different regions of the ovine fetal brain. (PDF 51 KB)

Additional file 2: Figure S2: Weighted gene co-expression network analysis (WGCNA) of the ovine fetal brain. WGCNA identifies modules of co-expressed genes following an increasing or decreasing expression temporal pattern during last stage of gestation in ovine fetal cortex **(A)**, brainstem **(B)**, hippocampus **(C)** and hypothalamus **(D)**. Modules are colored according the number of genes contained in the module. For example, the module’s color is turquoise if contains the largest number of genes. (PDF 205 KB)

Additional file 3: Table S1: Modules attributes for each brain region. Modules were determined by weighted gene-coexpression analysis on each brain region of the ovine fetal brain. Gene significance (GS) with the temporal pattern (Gestational Age) and module membership (MM) on each module (and respective p-value) was determined for each differentially expressed gene. (XLSX 4 MB)

Additional file 4: Figure S3: Relationship between modules identified in weighted gene co-expression networks and fetal age. The modules were determined in fetal cortex **(A)**, brainstem **(B)**, hippocampus (C) and hypothalamus **(D)**. Positive correlations are showed in red and negative correlations in blue. The stronger the color the higher the correlation index. (PDF 146 KB)

Additional file 5: Figure S4: Module membership versus gene significance plots. Modules composed by highly connected genes with the highest positive or negative correlation with gestational age and the respective dendrogram, identified en network analysis from the ovine fetal cortex **(A)**, brainstem **(B)**, hippocampus **(C)** and hypothalamus **(D)**. Each plot is colored according the corresponding module. (PDF 956 KB)

Additional file 6: Table S2: Enriched biological processes (BP) in the fetal brain during the last stage of gestation. These BP were significantly enriched with the co-expressed genes forming part of the top modules with highest positive correlation with fetal age in each of the ovine fetal brain regions (i.e., strongly increasing in expression toward the first day of extra-uterine life). (XLSX 12 KB)

Additional file 7: Figure S5: qRT-PCR validation for CD34, CD109, CD44, CD5 and CD9. Gene expression of CD34, CD109, CD44, CD5 and CD9 measured by microarray at 80, 100, 120, 130, 145 days of gestation and 1 day of extra-uterine life and corresponding fold changes in mRNA concentration relative to 80 days, measured by qRT- PCR in samples from ovine fetal brainsteam. Data are fold differences relative to mean expression at 80d. a - different from 80d values; b - different from 100d values; c - different from 120d values; d - different from 130d values; e - different from 145d values; f – different from 1d of extra-uterine life values. For all statistical comparisons, P < 0.05 was used as the criterion for significance. (PDF 402 KB)

Additional file 8: Figure S6: qRT-PCR validation for CSF1R, CSF1, IL34, CD11b and CD81. Gene expression of CSF1R, CSF1, IL34, CD11b and CD81 measured by microarray at 80, 100, 120, 130, 145 days of gestation and 1 day of extra-uterine life and corresponding fold changes in mRNA concentration relative to 80 days, measured by qRT- PCR in samples from ovine fetal cortex. Data are fold differences relative to mean expression at 80d. a - different from 80d values; b - different from 100d values; c - different from 120d values; d - different from 130d values; e - different from 145d values. For all statistical comparisons, P < 0.05 was used as the criterion for significance. (PDF 248 KB)

Additional file 9: Figure S7: qRT-PCR validation for CD3G, CD3D and CD3E. Gene expression of CD3G, CD3D and CD3E measured by microarray at 80, 100, 120, 130, 145 days of gestation and 1 day of extra-uterine life and corresponding fold changes in mRNA concentration relative to 80 days, measured by qRT- PCR in samples from ovine fetal hippocampus. Data are fold differences relative to mean expression at 80d. a - different from 80d values; b - different from 100d values; c - different from 120d values; d - different from 130d values; e - different from 145d values. For all statistical comparisons, P < 0.05 was used as the criterion for significance. (PDF 214 KB)

Additional file 10: Figure S8: qRT-PCR validation for MBP, CD24, FGRIIB, IL10, and TGFB. Gene expression of MBP **(A)**, CD24 **(B)** and FGRIIB, IL10, TGFB **(C)** measured by microarray at 80, 100, 120, 130, 145 days of gestation and 1 day of extra-uterine life and corresponding fold changes in mRNA concentration relative to 80 days, measured by qRT- PCR in samples from ovine fetal cortex. Data are fold differences relative to mean expression at 80d. a - different from 80d values; b - different from 100d values; c - different from 120d values; d - different from 130d values; e - different from 145d values. For all statistical comparisons, P < 0.05 was used as the criterion for significance. (PDF 271 KB)

Additional file 11: Table S3: Sequences of primers and probes for real-time PCR analysis. (DOC 38 KB)
