# Supplementary figures and images for: Transcriptomics of the late gestation ovine fetal brain: modeling the co-expression of immune marker genes
Source: BMC Genomics. 2014 Nov 19;15(1):1001. doi: 10.1186/1471-2164-15-1001 (PMC4253626; doi:10.1186/1471-2164-15-1001)

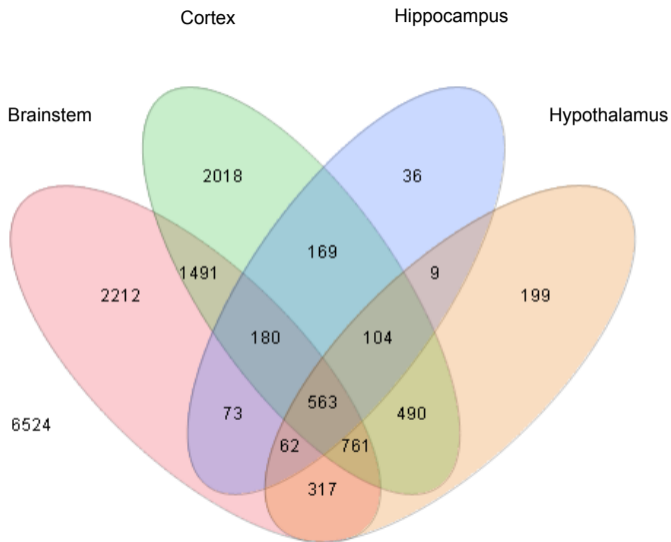

Supplement: Supplementary file 1 — Additional file 1: Figure S1: Venn Diagram of the number of differentially expressed genes (DEG) in the ovine fetal brain. DEG were selected as those following a temporal profile during the last stage of gestation in different regions of the ovine fetal brain. (PDF 51 KB) [file 12864_2014_6699_MOESM1_ESM.pdf]

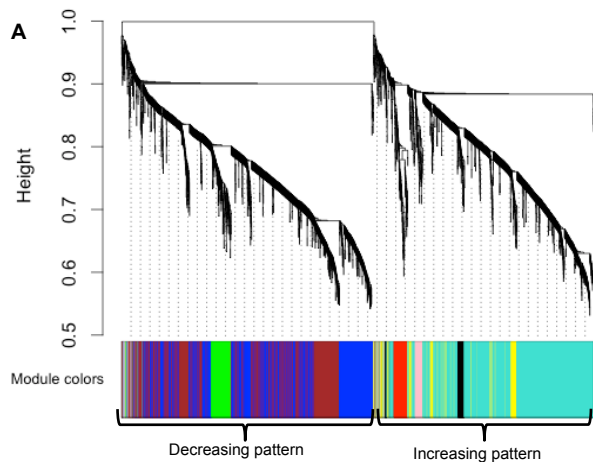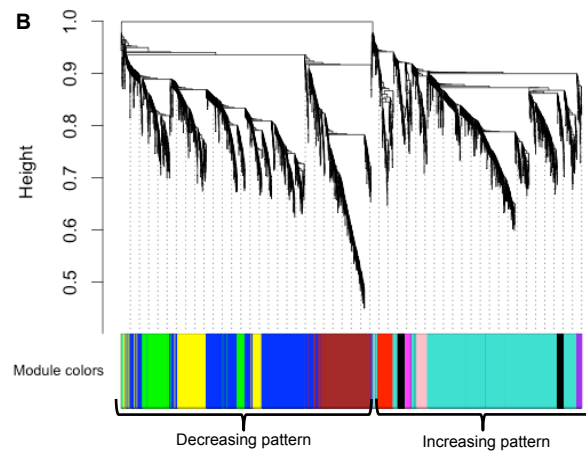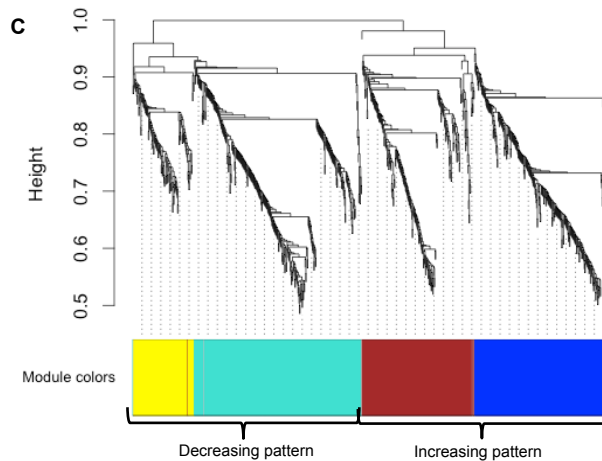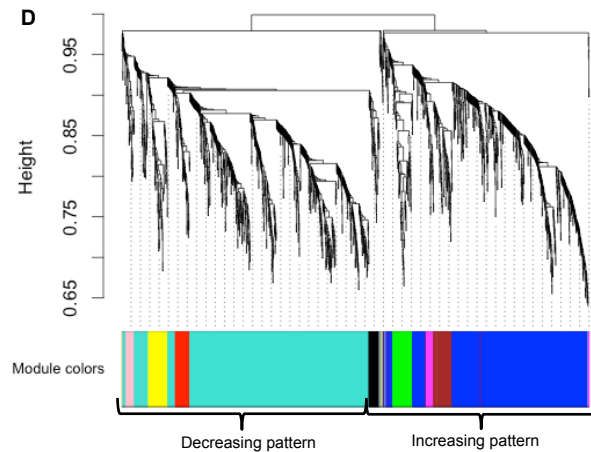

Supplement: Supplementary file 2 — Additional file 2: Figure S2: Weighted gene co-expression network analysis (WGCNA) of the ovine fetal brain. WGCNA identifies modules of co-expressed genes following an increasing or decreasing expression temporal pattern during last stage of gestation in ovine fetal cortex (A), brainstem (B), hippocampus (C) and hypothalamus (D). Modules are colored according the number of genes contained in the module. For example, the module’s color is turquoise if contains the largest number of genes. (PDF 205 KB) [file 12864_2014_6699_MOESM2_ESM.pdf]

**A**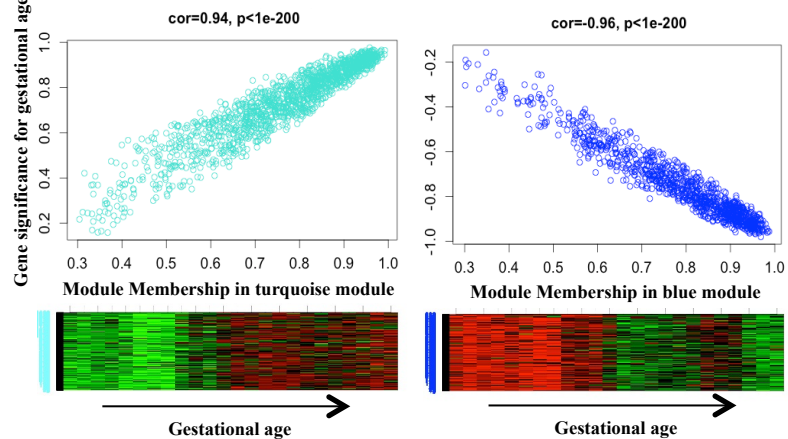**B**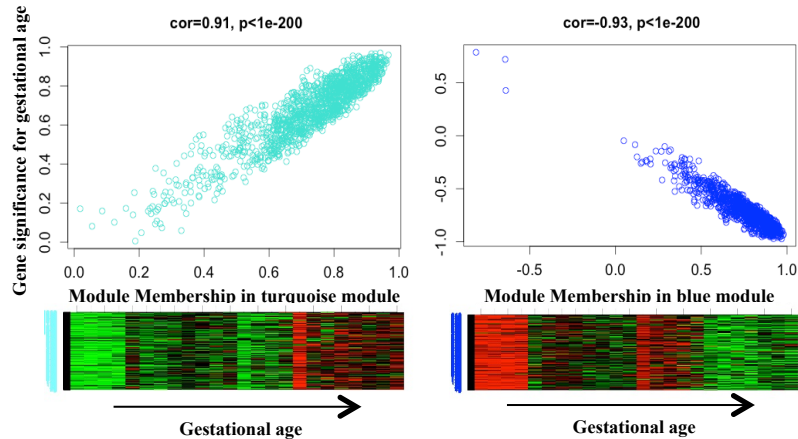**C**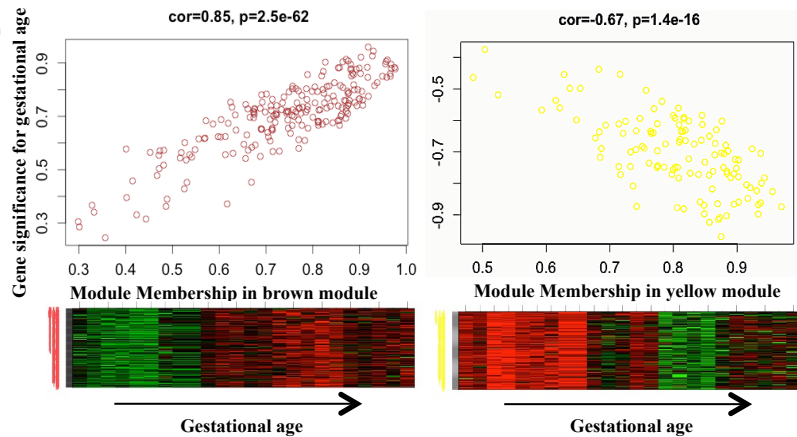**D**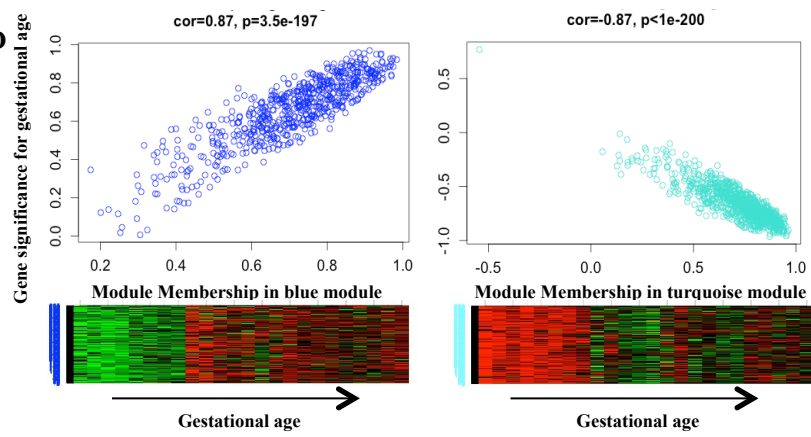

Supplement: Supplementary file 5 — Additional file 5: Figure S4: Module membership versus gene significance plots. Modules composed by highly connected genes with the highest positive or negative correlation with gestational age and the respective dendrogram, identified en network analysis from the ovine fetal cortex (A), brainstem (B), hippocampus (C) and hypothalamus (D). Each plot is colored according the corresponding module. (PDF 956 KB) [file 12864_2014_6699_MOESM5_ESM.pdf]

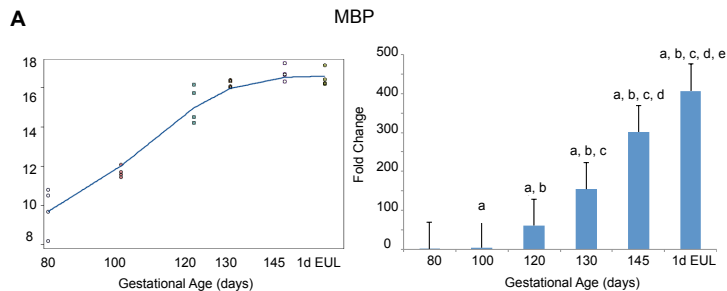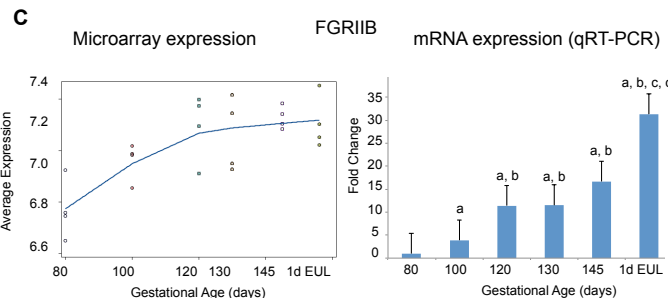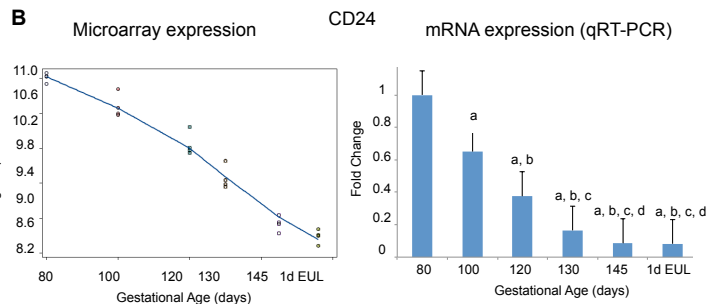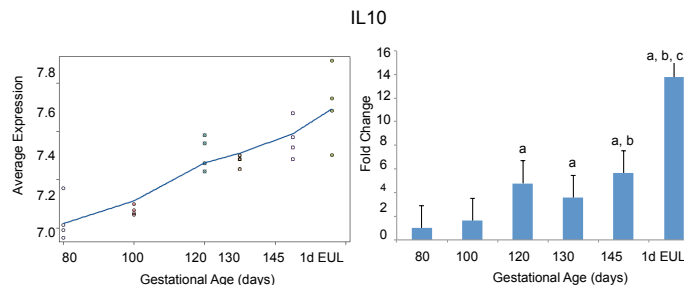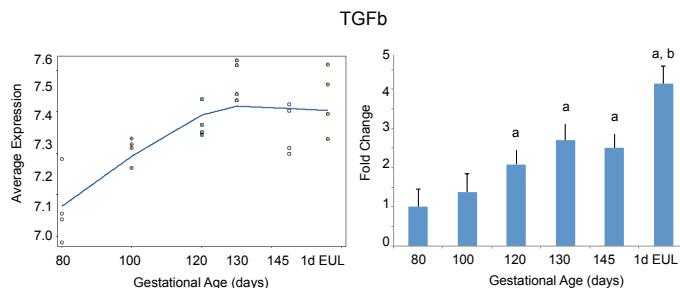

Supplement: Supplementary file 10 — Additional file 10: Figure S8: qRT-PCR validation for MBP, CD24, FGRIIB, IL10, and TGFB. Gene expression of MBP (A), CD24 (B) and FGRIIB, IL10, TGFB (C) measured by microarray at 80, 100, 120, 130, 145 days of gestation and 1 day of extra-uterine life and corresponding fold changes in mRNA concentration relative to 80 days, measured by qRT- PCR in samples from ovine fetal cortex. Data are fold differences relative to mean expression at 80d. a - different from 80d values; b - different from 100d values; c - different from 120d values; d - different from 130d values; e - different from 145d values. For all statistical comparisons, P < 0.05 was used as the criterion for significance. (PDF 271 KB) [file 12864_2014_6699_MOESM10_ESM.pdf]
