# Supplementary material for: Transcriptomics of the late gestation ovine fetal brain: modeling the co-expression of immune marker genes
Source: BMC Genomics. 2014 Nov 19;15(1):1001. doi: 10.1186/1471-2164-15-1001 (PMC4253626; doi:10.1186/1471-2164-15-1001)
Supplement: Supplementary file 4 — Additional file 4: Figure S3: Relationship between modules identified in weighted gene co-expression networks and fetal age. The modules were determined in fetal cortex (A), brainstem (B), hippocampus (C) and hypothalamus (D). Positive correlations are showed in red and negative correlations in blue. The stronger the color the higher the correlation index. (PDF 146 KB) [file 12864_2014_6699_MOESM4_ESM.pdf]

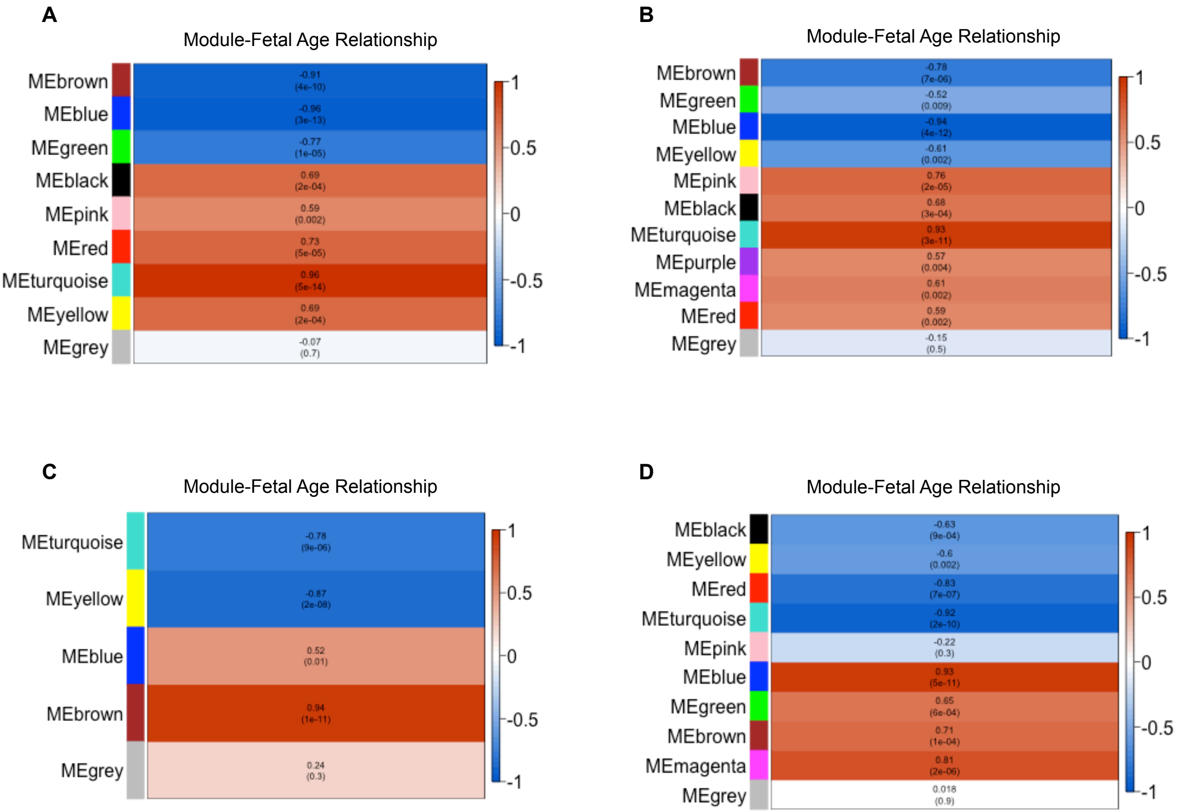
A
Module-Fetal Age Relationship
MEbrown
MEblue
MEgreen
MEblack
MEpink
MEred
MEturquoise
MEyellow
MEgrey
-0.91 (4e-10)
-0.96 (3e-13)
-0.77 (1e-05)
0.69 (2e-04)
0.59 (0.002)
0.73 (5e-05)
0.96 (5e-14)
0.69 (2e-04)
-0.07 (0.7)
1
0.5
0
-0.5
-1
B
Module-Fetal Age Relationship
MEbrown
MEgreen
MEblue
MEyellow
MEpink
MEblack
MEturquoise
MEpurple
MEmagenta
MEred
MEgrey
-0.78 (7e-06)
-0.52 (0.009)
-0.94 (4e-12)
-0.61 (0.002)
0.76 (2e-05)
0.68 (3e-04)
0.93 (3e-11)
0.57 (0.004)
0.61 (0.002)
0.59 (0.002)
-0.15 (0.5)
1
0.5
0
-0.5
-1
C
Module-Fetal Age Relationship
MEturquoise
MEyellow
MEblue
MEbrown
MEgrey
-0.78 (9e-06)
-0.87 (2e-08)
0.52 (0.01)
0.94 (1e-11)
0.24 (0.3)
1
0.5
0
-0.5
-1
D
Module-Fetal Age Relationship
MEblack
MEyellow
MEred
MEturquoise
MEpink
MEblue
MEgreen
MEbrown
MEmagenta
MEgrey
-0.63 (9e-04)
-0.6 (0.002)
-0.83 (7e-07)
-0.92 (2e-10)
-0.22 (0.3)
0.93 (5e-11)
0.65 (6e-04)
0.71 (1e-04)
0.81 (2e-06)
0.018 (0.9)
1
0.5
0
-0.5
-1
