# Supplementary material for: Transcriptomics of the late gestation ovine fetal brain: modeling the co-expression of immune marker genes
Source: BMC Genomics. 2014 Nov 19;15(1):1001. doi: 10.1186/1471-2164-15-1001 (PMC4253626; doi:10.1186/1471-2164-15-1001)
Supplement: Supplementary file 7 — Additional file 7: Figure S5: qRT-PCR validation for CD34, CD109, CD44, CD5 and CD9. Gene expression of CD34, CD109, CD44, CD5 and CD9 measured by microarray at 80, 100, 120, 130, 145 days of gestation and 1 day of extra-uterine life and corresponding fold changes in mRNA concentration relative to 80 days, measured by qRT- PCR in samples from ovine fetal brainsteam. Data are fold differences relative to mean expression at 80d. a - different from 80d values; b - different from 100d values; c - different from 120d values; d - different from 130d values; e - different from 145d values; f – different from 1d of extra-uterine life values. For all statistical comparisons, P < 0.05 was used as the criterion for significance. (PDF 402 KB) [file 12864_2014_6699_MOESM7_ESM.pdf]

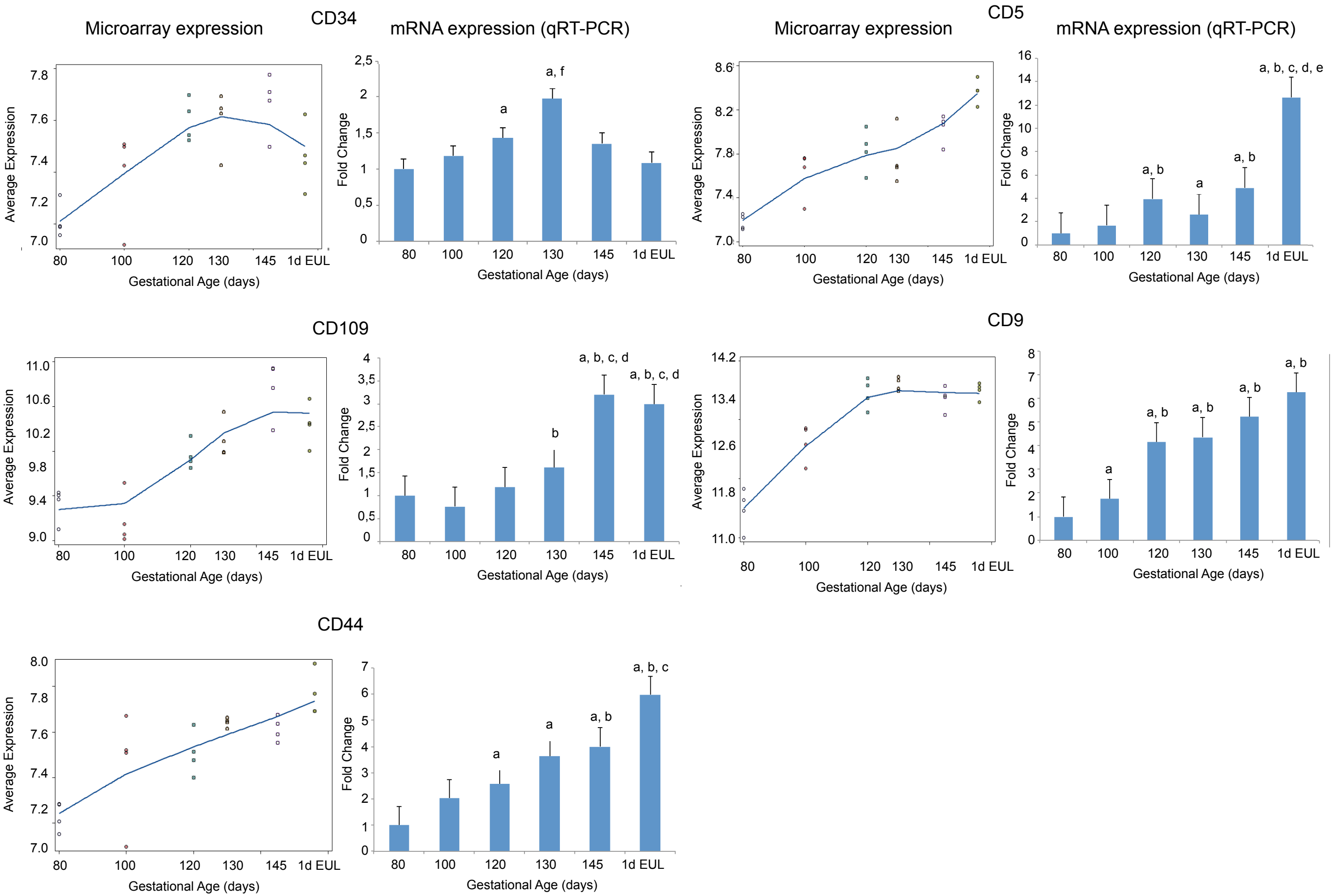

CD34
Microarray expression
mRNA expression (qRT-PCR)
Average Expression
7.8
7.6
7.4
7.2
7.0
80
100
120
130
145
1d EUL
Gestational Age (days)
Fold Change
2,5
2
1,5
1
0,5
0
a
a, f
CD5
Microarray expression
mRNA expression (qRT-PCR)
8.6
8.2
7.8
7.4
7.0
16
14
12
10
8
6
4
2
0
a, b
a
a, b
a, b, c, d, e
CD109
11.0
10.6
10.2
9.8
9.4
9.0
4
3,5
3
2,5
2
1,5
1
0,5
0
b
a, b, c, d
a, b, c, d
CD9
14.2
13.4
12.6
11.8
11.0
8
7
6
5
4
3
2
1
0
a
a, b
a, b
a, b
a, b
CD44
8.0
7.8
7.6
7.4
7.2
7.0
7
6
5
4
3
2
1
0
a
a
a, b
a, b, c
