# Supplementary material for: Transcriptomics of the late gestation ovine fetal brain: modeling the co-expression of immune marker genes
Source: BMC Genomics. 2014 Nov 19;15(1):1001. doi: 10.1186/1471-2164-15-1001 (PMC4253626; doi:10.1186/1471-2164-15-1001)
Supplement: Supplementary file 8 — Additional file 8: Figure S6: qRT-PCR validation for CSF1R, CSF1, IL34, CD11b and CD81. Gene expression of CSF1R, CSF1, IL34, CD11b and CD81 measured by microarray at 80, 100, 120, 130, 145 days of gestation and 1 day of extra-uterine life and corresponding fold changes in mRNA concentration relative to 80 days, measured by qRT- PCR in samples from ovine fetal cortex. Data are fold differences relative to mean expression at 80d. a - different from 80d values; b - different from 100d values; c - different from 120d values; d - different from 130d values; e - different from 145d values. For all statistical comparisons, P < 0.05 was used as the criterion for significance. (PDF 248 KB) [file 12864_2014_6699_MOESM8_ESM.pdf]

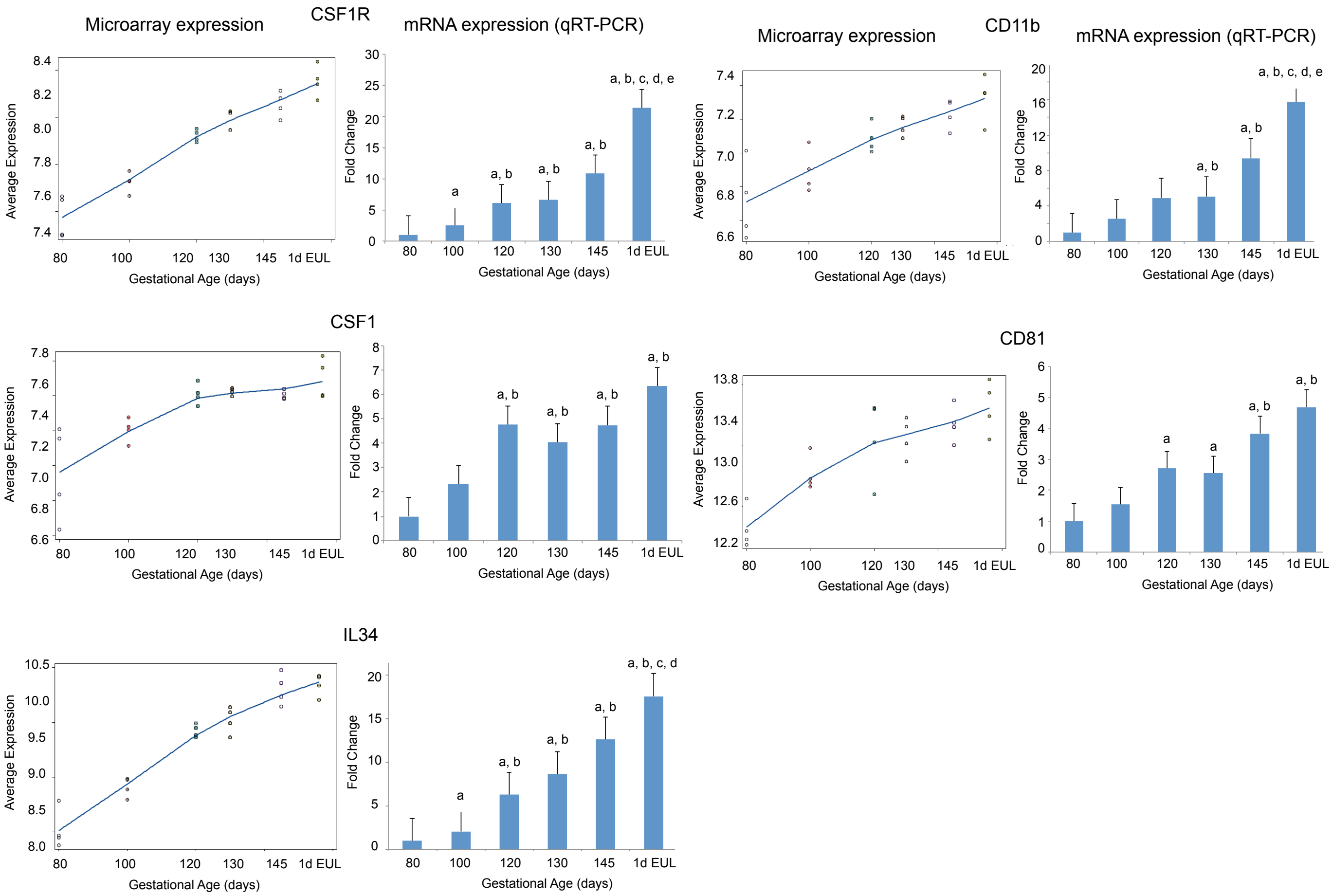

CSF1R
Microarray expression
mRNA expression (qRT-PCR)
a
a, b
a, b
a, b
a, b, c, d, e
CD11b
Microarray expression
mRNA expression (qRT-PCR)
a, b
a, b
a, b, c, d, e
CSF1
a, b
a, b
a, b
a, b
CD81
a
a
a, b
a, b
IL34
a
a, b
a, b
a, b
a, b, c, d
Average Expression
Fold Change
80
100
120
130
145
1d EUL
Gestational Age (days)
