# Supplementary material for: Transcriptomics of the late gestation ovine fetal brain: modeling the co-expression of immune marker genes
Source: BMC Genomics. 2014 Nov 19;15(1):1001. doi: 10.1186/1471-2164-15-1001 (PMC4253626; doi:10.1186/1471-2164-15-1001)
Supplement: Supplementary file 9 — Additional file 9: Figure S7: qRT-PCR validation for CD3G, CD3D and CD3E. Gene expression of CD3G, CD3D and CD3E measured by microarray at 80, 100, 120, 130, 145 days of gestation and 1 day of extra-uterine life and corresponding fold changes in mRNA concentration relative to 80 days, measured by qRT- PCR in samples from ovine fetal hippocampus. Data are fold differences relative to mean expression at 80d. a - different from 80d values; b - different from 100d values; c - different from 120d values; d - different from 130d values; e - different from 145d values. For all statistical comparisons, P < 0.05 was used as the criterion for significance. (PDF 214 KB) [file 12864_2014_6699_MOESM9_ESM.pdf]

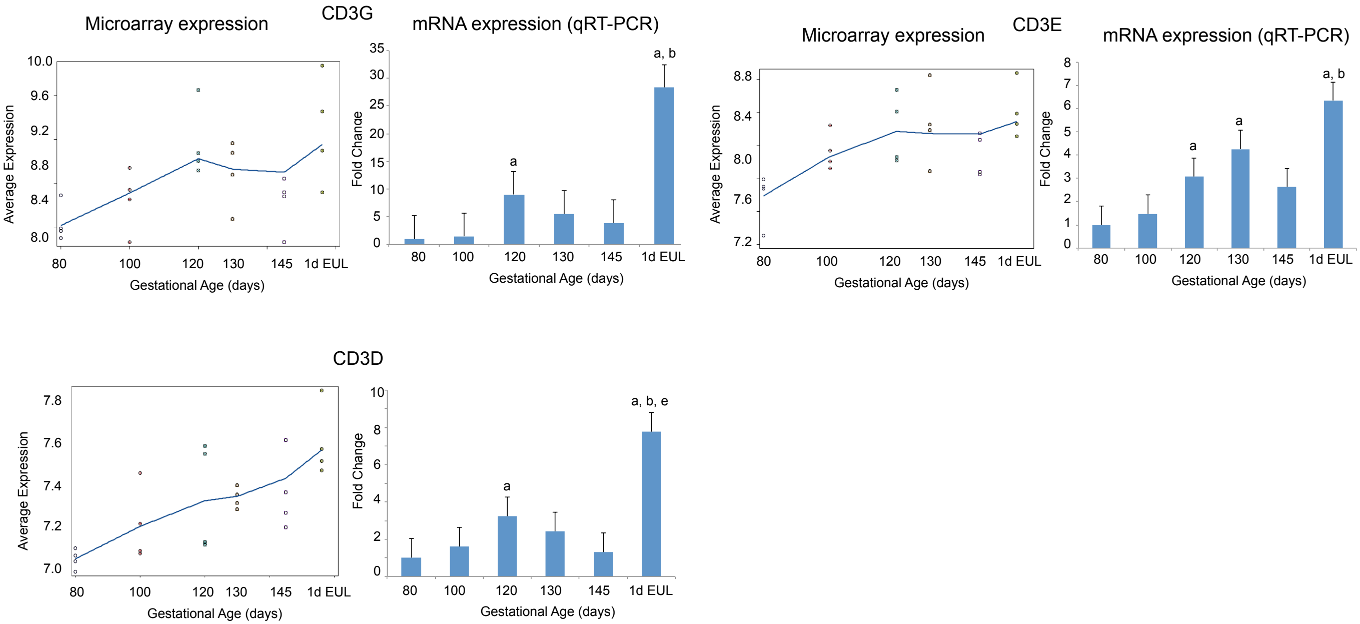
CD3G
Microarray expression
mRNA expression (qRT-PCR)
Average Expression
10.0
9.6
9.2
8.8
8.4
8.0
80
100
120
130
145
1d EUL
Gestational Age (days)
Fold Change
35
30
25
20
15
10
5
0
a
a, b
CD3E
Microarray expression
mRNA expression (qRT-PCR)
8.8
8.4
8.0
7.6
7.2
8
7
6
5
4
3
2
1
0
CD3D
7.8
7.6
7.4
7.2
7.0
a, b, e
