# Supplementary material for: Transcriptomics of the late gestation ovine fetal brain: modeling the co-expression of immune marker genes
Source: BMC Genomics. 2014 Nov 19;15(1):1001. doi: 10.1186/1471-2164-15-1001 (PMC4253626; doi:10.1186/1471-2164-15-1001)
Supplement: Supplementary file 11 — Additional file 11: Table S3: Sequences of primers and probes for real-time PCR analysis. (DOC 38 KB) [file 12864_2014_6699_MOESM11_ESM.doc]

### Additional table 3 – Sequences of primers and probes for real-time PCR analysis

| **Gene** | **forward primer** | **reverse primer** | **accession #** |
| --- | --- | --- | --- |
| **oCD34** | TGGAGCCGTGAACTCTTCTGT | CACAAAGCTGATGAGGGTAGAAGAC | XM_004014117 |
| **bCD109** | CACAAGATGCTTCAGTGTCCATAGT | CTGCGCTCTGGAGTTGTAGCT | XM_002690022 |
| **bCD44** | CGGGTTCATAGAAGGGCATGT | TGTTCGCAGCACAGATGGA | NM_174013 |
| **oCD5** | CAGTGTGGCTCCTTCCTGAAG | TTTGGCCTCCTGGCTTTG | NM_173899 |
| **oCD9** | CTGAAGCCATCGACGAGATCT | CCACGGCAATCCCAATACC | NM_001114764 |
| **oCD3G** | ATTGCTGGACAGGAAGGAGTTC | TGGTAGAGCTGGTCATTGTTCAA | X52994 |
| **oCD3D** | ATCGAATGTGCCAGAACTGTGT | GGCAATGATGTCGGTGATGA | NM_001009382 |
| **oCD3E** | GAGGTGGCCACAATCATCGT | TTTCGGCTCTTGCTCCAGTAA | NM_001009418 |
| **bCSF1** | GACTGGAACATTTTCAGCAAGAACT | TCAGGCTTGGTCACCACATC | NM_174026 |
| **bCSF1R** | ACACAAAACTCGCAATCTCTCAAC | TCGAGTTCGAGAGTCAGGACTTT | NM_001075403 |
| **bIL34** | GATTCCTGCGGGACAAGCT | CACCCCCTCATAAGGCACACT | NM_001100324 |
| **oCD11b** | CTCCCTCTGCTCCGTGGAT | TCGCATCTGCTCATAAAAATGG | NM_001082593 |
| **oCD81** | CCTCCTGTATCTGGAGCTTGGA | CAATAAGGATGTAGATGCCCACATA | NM_001127281 |
| **oFCGR2b** | CTGCAGTGGCTGTTGTTGCT | CGGCTGAAATTGGCTTTCTC | NM_001139453 |
| **oIL10** | CCAGGATGGTGACTCGACTAGAC | TGGCTCTGCTCTCCCAGAAC | NM_001009327 |
| **oTGFb** | CAGTAAGGATAACACGCTTCAAGTG | CCGGTTCATGCCGTGAAT | NM_001009400 |
| **bCD24** | GCCCCTCATCCAGCCAAT | GACTGGCTGTTGACTGCAGAGT | GJ060586 |
| **bMBP** | AAAACCCTGTAGTGCACTTCTTCA | CCCTTTCCTTGCGATGGA | NM_001206674 |
